# Antiseptic Functions of CGK012 against HMGB1-Mediated Septic Responses

**DOI:** 10.3390/ijms25052976

**Published:** 2024-03-04

**Authors:** Yun Jin Park, Jong Beom Heo, Yoon-Jung Choi, Sanghee Cho, Taeho Lee, Gyu Yong Song, Jong-Sup Bae

**Affiliations:** 1 Research Institute of Pharmaceutical Sciences, College of Pharmacy, Kyungpook National University, Daegu 41566, Republic of Korea; dbswls101@naver.com (Y.J.P.); sanghee3472@naver.com (S.C.); tlee@knu.ac.kr (T.L.); 2 College of Pharmacy, Chungnam National University, Daejon 34134, Republic of Korea; songmeom@gmail.com (J.B.H.); cyjjh0609@naver.com (Y.-J.C.); 3 AREZ Co., Ltd., Daejeon 34036, Republic of Korea

**Keywords:** CGK012, sepsis, HMGB1, endothelium

## Abstract

High mobility group box 1 (HMGB1), a protein with important functions, has been recognized as a potential therapeutic target for the treatment of sepsis. One possible mechanism for this is that inhibiting HMGB1 secretion can exert antiseptic effects, which can restore the integrity of the vascular barrier. (7S)-(+)-cyclopentyl carbamic acid 8,8-dimethyl-2-oxo-6,7-dihydro-2H,8H-pyrano[3,2-g]chromen-7-yl-ester (CGK012) is a newly synthesized pyranocoumarin compound that could function as a novel small-molecule inhibitor of the Wnt/β-catenin signaling pathway. However, no studies have yet determined the effects of CGK012 on sepsis. We investigated the potential of CGK012 to attenuate the excessive permeability induced by HMGB1 and enhance survival rates in a mouse model of sepsis with reduced HMGB1 levels following lipopolysaccharide (LPS) treatment. In both LPS-stimulated human endothelial cells and a mouse model exhibiting septic symptoms due to cecal ligation and puncture (CLP), we assessed proinflammatory protein levels and tissue damage biomarkers as indicators of reduced vascular permeability. CGK012 was applied after induction in human endothelial cells exposed to LPS and the CLP-induced mouse model of sepsis. CGK012 effectively mitigated excessive permeability and suppressed HMGB1 release, resulting in improved vascular stability, decreased mortality, and enhanced histological conditions in the mouse model of CLP-induced sepsis. In conclusion, our findings indicate that CGK012 treatment in mice with CLP-induced sepsis diminished HMGB1 release and increased the survival rate, suggesting its potential as a pharmaceutical intervention for sepsis.

## 1. Introduction

Sepsis is a serious and often deadly infection that remains difficult to treat despite recent research efforts owing to its ability to cause uncontrollable immune activity and inflammation, which lead to high mortality and morbidity rates [1]. The main cause of death is multi-organ failure (MOF) rather than oxygen shortage, with kidney dysfunction being a significant contributor to MOF, which affects various systems [2,3]. The mechanisms and pathological characteristics of sepsis have yet to be fully understood; however, antigen-derived molecules trigger the release of inflammatory factors from macrophages and leukocytes, including early-phase mediators, such as interleukin (IL)-1β and tumor necrosis factor (TNF)-α, and late-phase factors, such as high mobility group box 1 (HMGB1) [4,5]. Lipopolysaccharide (LPS) is a glycolipid that is used to induce vascular damage in both in vivo and in vitro studies [6], whereas HMGB1 is a significant factor in sepsis and remains stable at high levels for up to 1–1.5 days in animal models [7,8]. Inhibiting the secretion of HMGB1 from damaged cells and stimulated immunocytes may therefore be a promising strategy for the treatment of lethal septic diseases. The latest clinical guidelines for sepsis management emphasize early recognition and intervention, prompt administration of appropriate antibiotics, fluid resuscitation, and hemodynamic support to optimize patient outcomes [9]. These guidelines stress the importance of timely assessment and risk stratification, along with close monitoring of vital signs and laboratory parameters. Additionally, they advocate for the use of bundled care approaches, such as the “sepsis bundle” or “sepsis protocol”, which outline specific interventions to be implemented within a defined timeframe [9]. Furthermore, the guidelines highlight the importance of source control, adequate organ support, and ongoing reassessment to guide therapy adjustments. Overall, the current standards of sepsis care prioritize a multidisciplinary, evidence-based approach aimed at improving patient survival and reducing morbidity associated with septic shock and organ dysfunction [9]. During sepsis, several inflammatory biomarkers play crucial roles in diagnosis, prognosis, and guiding treatment decisions. These biomarkers include C-reactive protein (CRP), procalcitonin (PCT), interleukin-6 (IL-6), interleukin-10 (IL-10), and TNF-α [1,2,3,9]. CRP and PCT have been commonly used to assess the severity of systemic inflammation, with elevated levels correlating with the presence and severity of infection. IL-6 is a key mediator of the inflammatory response and is often elevated in sepsis, reflecting the extent of cytokine activation. IL-10 acts as an anti-inflammatory cytokine, and its levels may indicate the balance between pro- and anti-inflammatory responses to sepsis. TNF-α is another proinflammatory cytokine that contributes to the systemic inflammatory cascade in sepsis. Monitoring these biomarkers can aid in the early detection of sepsis, risk stratification, and assessment of treatment response, thereby guiding clinical management and improving patient outcomes.

**Figure 1:**
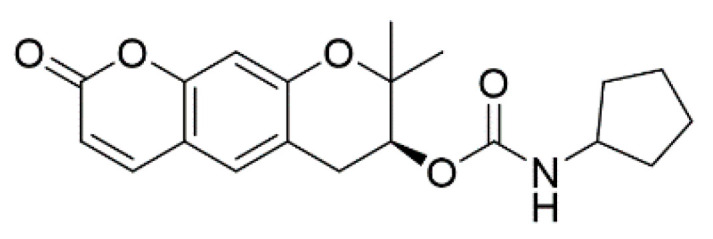
Chemical structure of CGK012.

Wnt/β-catenin signaling pathway activation has been associated with HMGB1 expression and secretion in intestinal tumorigenesis [10]. (7S)-(+)-cyclopentyl carbamic acid 8,8-dimethyl-2-oxo-6,7-dihydro-2H,8H-pyrano[3,2-g]chromen-7-yl-ester (CGK012, Figure 1) is a newly synthesized pyranocoumarin substance that suppresses the activation and transcription of β-catenin related to Wnt3a-CM [11]. Evidence shows that CGK012 attenuated Wnt/β-catenin signaling by preventing the proliferation of multiple myeloma cells through the downregulation of intracellular β-catenin [11]. CGK012 induced β-catenin phosphorylation at Ser33/Ser37/Th41 through a mechanism independent of glycogen synthase kinase-3β (GSK-3β), causing proteasomal degradation and reducing intracellular β-catenin levels. Furthermore, CGK012 consistently decreased the amount of β-catenin and repressed the expression of cyclin D1, c-myc, and axin-2 (downstream target genes of β-catenin) in RPMI-8226 multiple myeloma cells [11]. Nevertheless, the mechanism by which CGK012 is involved in sepsis and systemic inflammation mediated by HMGB1 still remains unknown. Thus, we aimed to demonstrate the effectiveness of CGK012 in preventing and treating HMGB1-mediated vascular integrity failure in vitro and in vivo.

## 2. Results

### 2.1. CGK012 Inhibits HMGB1 Release in the LPS-Stimulated Human Umbilical Vein Endothelial Cells (HUVECs) and Cecal Ligation and Puncture (CLP)-Induced Sepsis Mouse Model

**Figure 2:**
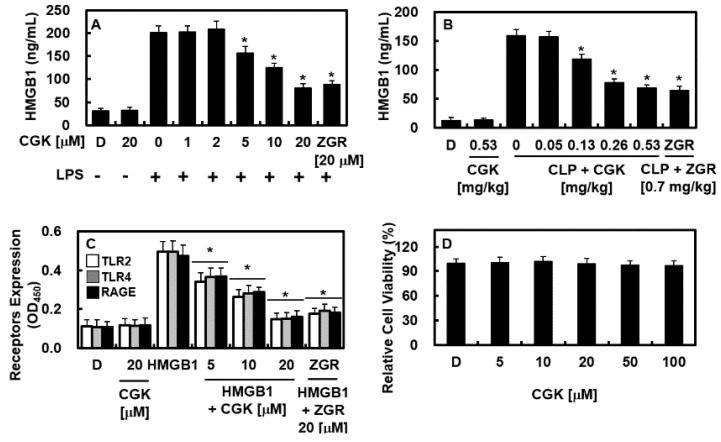
Effects of CGK012 on HMGB1 release and HMGB1 receptor expression. The effects of CGK012 on HMGB1 release in LPS-activated HUVECs (**A**) and mice subjected to CLP surgery (**B**) were measured using an ELISA. The expression levels of TLR2, TLR4, and RAGE by HMGB1 in response to CGK012 treatment were measured (**C**). The MTT assay was used to evaluate cellular viability after treatment with LPS or CGK012 (**D**). Data are the mean ± SD values from three independent experiments conducted in triplicate on different days. The vehicle control for the study was 0.2% dimethyl sulfoxide (**D**). Our results indicate that CGK012 treatment reduced HMGB1 release, improved vascular wall stability, and decreased HMGB1 receptor expression. * *p* < 0.01 versus LPS alone (**A**), CLP alone (**B**), or HMGB1 alone (**C**).

We determined whether LPS-induced HMGB1 secretion was modulated by CGK012 administration. First, we applied LPS to activate HUVECs and then administered CGK012 at doses of 1, 2, 5, 10, or 20 μM or zingerone (ZGR). HMGB1 release from cells was detected using an enzyme-linked immunosorbent assay (ELISA). Our results showed that HUVECs treated with LPS showed a considerable increase in HMGB1 release. In contrast, CGK012-treated cells showed lower HMGB1 release after LPS treatment (Figure 2A). The positive control compound ZGR was applied to induce an ameliorative effect on inflammation [12,13,14], similar to CGK012 treatment. To confirm the effects of CGK012 in vivo, the mice were injected with CGK012 or ZGR intravenously at 12 h after CLP induction and then euthanized 12 h later. HMGB1 release induced by CLP was remarkably decreased in CGK012-treated mice (Figure 2B). Moreover, we found that CGK012 decreased the expression of pathogen-associated molecular patterns, such as toll-like receptor (TLR)2, TLR4, and the HMGB1 receptor protein receptor for advanced glycation end product (RAGE), in HUVECs (Figure 2C). The 3-(4,5-dimethylthiazol-2-yl)-2,5-diphenyltetrazolium bromide (MTT) assay was used to detect cell viability after the CGK012 treatment of HUVECs. CGK012 treatment at various doses (5, 10, 20, 50, or 100 μM) for 48 h had no effect on cell viability (Figure 2D). Thus, our results indicate that CGK012 may be a promising agent for modulating the early phase of sepsis by inhibiting HMGB1 secretion.

### 2.2. Effects of CGK012 on the Activation of Silent Information Regulator Sirtuin 1 (SIRT1) and HMGB1 Acetylation

**Figure 3:**
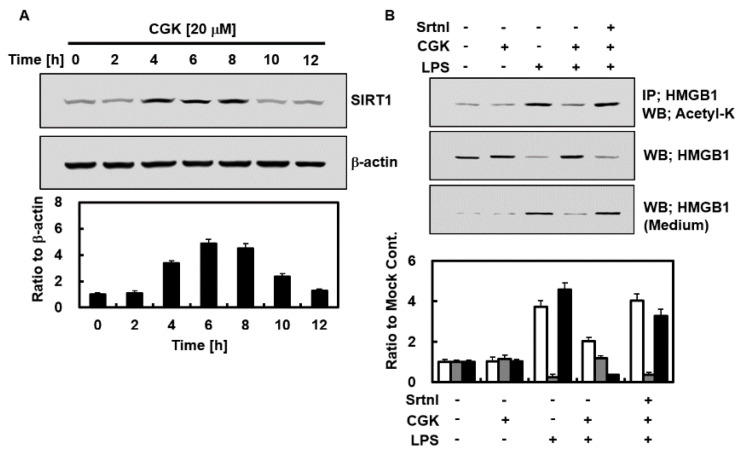
Effects of CGK012 on SIRT1 activation and HMGB1 acetylation. In part (**A**), cells were treated with CGK012 across various time intervals (0–12 h), after which SIRT1 expression levels were measured using Western blotting. Part (**B**) determined the effects of CGK012 on HMGB1 acetylation and SIRT1 expression in HUVECs. The cells were treated with LPS alone or with CGK012 and an SIRT1 inhibitor (sirtinol) before CGK012 treatment. After incubation, the cells were lysed for immunoprecipitation. The immunoprecipitated cell lysates were analyzed for HMGB1 acetylation and the total HMGB1 protein level using anti-acetyl-lysine and anti-HMGB1 antibodies, respectively. In addition, the released HMGB1 was detected in the medium using Western blotting after 16 h of incubation. Below: Densitometric intensities were normalized to β-actin (**A**) or mock control (**B**).

Excessive HMGB1 acetylation affects its binding capacity to DNA or cellular plasma [15]. The hyperacetylation of serine residues in HMGB1 hinders nuclear migration and facilitates cytoplasmic migration [16]. Conversely, SIRT1 stimulates the deacetylation of HMGB1, making it a potential deacetylation target [17]. Therefore, we explored the effects of CGK012 on SIRT1 expression. We found that SIRT1 was highly expressed after 4 h of culture, peaking at 6 h, remaining constant until 8 h, and disappearing at 12 h (Figure 3A). To investigate how CGK012 inhibited LPS-induced HMGB1 release, we assessed whether CGK012 affected HMGB1 deacetylation and SIRT1 expression. Notably, CGK012 treatment substantially reduced LPS-induced acetylation of HMGB1 (Figure 3B). To confirm the role of SIRT1 in suppressing HMGB1 secretion through HMGB1 deacetylation, we evaluated the effects of the SIRT1 repressor on HMGB1 secretion. Our results revealed that sirtinol stimulation significantly altered the effects of CGK012 (Figure 3B) and promoted an increase in acetylation and the subsequent release of HMGB1. Overall, these findings suggest that CGK012 significantly reduces HMGB1 secretion in LPS-stimulated HUVECs via the SIRT1-related deacetylation of HMGB1.

### 2.3. CGK012 Inhibits HMGB1-Mediated Disruption of the Vascular Barrier Integrity

**Figure 4:**
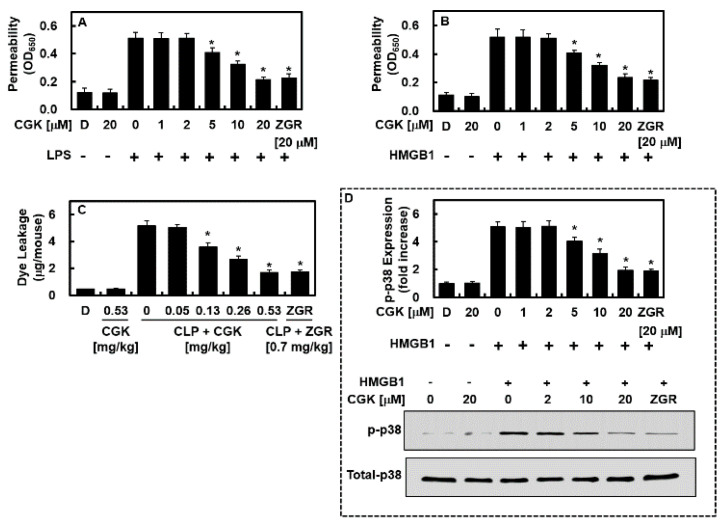
Effects of CGK012 on HMGB1-induced permeability in vitro and in vivo. The effects of CGK012 on LPS- (100 ng/mL) (**A**), HMGB1- (1 μg/mL) (**B**) or CLP- (**C**) induced hyperpermeability in HUVECs (**A**,**B**) or mice (**C**) (*n* = 5). (**D**) The effects of CGK012 on the HMGB1-mediated phosphorylation of p38 were determined using an ELISA (upper image) or Western blotting (lower image). Data are expressed as the mean ± SD values of three independent experiments on different days. * *p* < 0.01 versus treated with LPS (**A**) or HMGB1 (**B**–**D**).

To determine the therapeutic potential of CGK012 on the cohesiveness of the vascular barrier integrity, which is essential for pathological vascular inflammation, we analyzed the vascular permeability of HUVECs, representing the status of the vascular barrier integrity, given that LPS and HMGB1 can decrease vascular barrier cohesiveness. LPS-stimulated or naïve HMGB1-treated HUVECs were treated with CGK012 or ZGR for 6 h, after which changes in barrier integrity were determined by measuring the leakage of albumin bound to Evans blue dye through a single layer of HUVECs. Notably, CGK012 inhibited the excessive permeability induced by LPS and HMGB1 (Figure 4A,B). In vivo experiments were also performed to investigate the barrier-stabilizing effects of CGK012. Mice were injected with CGK012 or ZGR 12 h after CLP induction, euthanized 12 h after the injection, and measured for the quantity of Evans blue dye in abdominal rinsed fluids. Treatment with CGK012 downregulated the excessive permeability induced by CLP (Figure 4C). Given that HMGB1-induced vascular injury is regulated by the phosphorylation of p38, we stimulated HUVECs with HMGB1 and treated them with CGK012 or ZGR for 6 h and then measured the effects of CGK012 or ZGR on p38 phosphorylation using an ELISA. Notably, HMGB1 increased the level of phosphorylated p38, whereas CGK012 inhibited it (Figure 4D). The downregulation of HMGB1-induced barrier integrity reduction, leakage, and phosphorylated p38 suggests that CGK012 may be a promising molecule for the treatment of sepsis.

### 2.4. Effects of CGK012 on HMGB1-Mediated Expression of CAMs, Adhesion of Neutrophils, and Migration of Leukocytes

**Figure 5:**
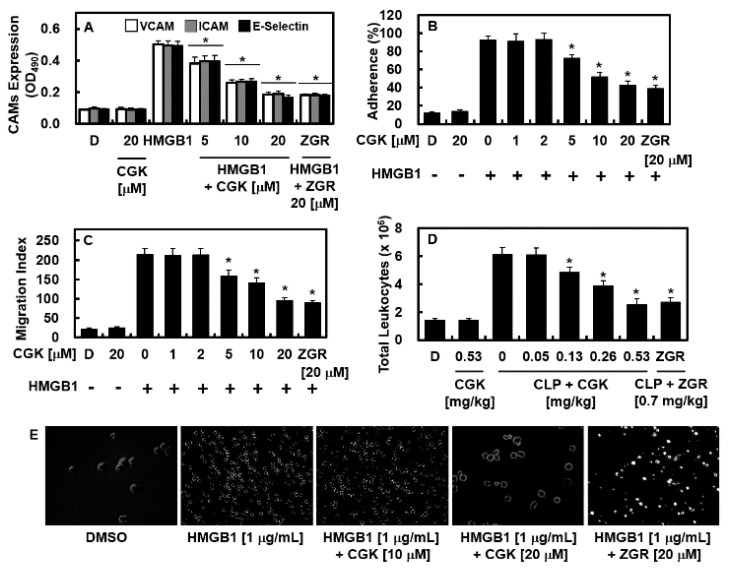
Effects of CGK012 on HMGB1-mediated proinflammatory responses. The effects of CGK012 on HMGB1-induced (**A**) expression levels of E-selectin (black bar), VCAM-1 (white bar), and ICAM-1 (gray bar) in HUVECs; (**B**,**E**) adherence of neutrophils to HUVEC monolayers; and (**C**) migration of neutrophils through HUVEC monolayers. (**D**) The effects of CGK012 on the CLP-induced migration of leukocytes into the peritoneal cavities of mice were analyzed by counting leukocytes in the peritoneal fluid. (**E**) Representative images for three independent experiments on different days with results similar to those in panel (**C**). Data are expressed as the mean ± SD values of three independent experiments on different days. * *p* < 0.01 versus treated with HMGB1 or CLP.

HMGB1 induces the expression of cellular adhesive molecules, such as E-selectin, ICAM-1, and VCAM-1, on the exposed part of endothelial cells to facilitate the migration of immune cells through the vascular endothelial cell layer to the site of inflammation. In light of this, we investigated the effects of HMGB1 on the expression of adhesion molecules in HUVECs, the adhesiveness of neutrophilic leukocytes, and their migration across single layers of HUVECs. We activated HUVECs with HMGB1 (1 μg/mL) for 16 h and then treated them with CGK012 or ZGR continuously for 6 h. Notably, CGK012 dose-dependently downregulated the production of adhesion molecules, which was otherwise increased by HMGB1 (Figure 5A). Moreover, CGK012 decreased not only the adhesiveness of human neutrophilic leukocytes to HUVECs but also their subsequent translocation (Figure 5B,C,E). The results of the in vivo assay were consistent with these findings, demonstrating a decrease in the HMGB1-mediated translocation of immune cells in the abdominal fluid (Figure 5B,C,E). Thus, our results indicate that CGK012 decreased the adhesion and migration of inflammatory immune cells induced by HMGB1 stimulation.

### 2.5. CGK012 Suppresses NF-κB/ERK Signaling and IL-1β, IL-6, and TNF-α Production

**Figure 6:**
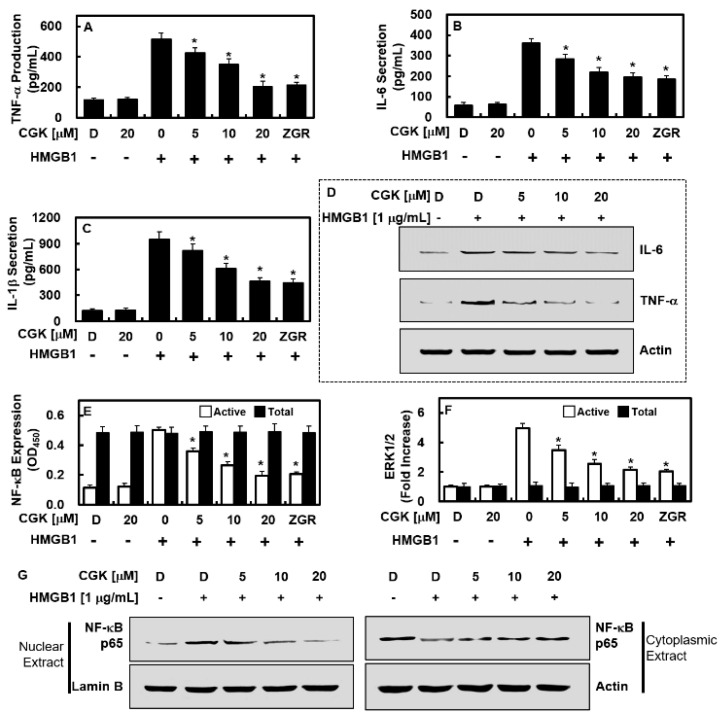
Effects of CGK012 on the HMGB1-induced production of IL-6, TNF-α, and IL-1β and the activation of the NF-κB/ERK1/2 pathways. The effects of CGK012 on the HMGB1-mediated production of TNF-α (**A**,**D**), IL-6 (**B**,**D**), and IL-1β (**C**) in the HUVECs were determined using ELISA (**A**–**C**) or Western blotting (**D**). (**E**,**F**) The effects of CGK012 on the HMGB1-mediated activation of (**E**) NF-κB p65 (white bar for phospho-p65 and black bar for total p65) and (**F**) ERK1/2 (white bar for phospho-ERK1/2 and black bar for total ERK1/2) in HUVECs. (**G**) Subcellular levels of NF-κB in nuclear and cytoplasmic fractions were evaluated via Western blotting using actin and lamin B as loading controls for the cytoplasmic and nuclear extracts, respectively. Data are expressed as the mean ± SD values of three independent experiments on different days. * *p* < 0.01 versus treatment with HMGB1.

HMGB1 has been known to induce the production of cytokines like TNF-α, IL-1β, and IL-6 through various mechanisms, including the NF-κB and ERK 1/2 pathways, exacerbating the pathological condition of sepsis. To investigate whether CGK012 can reduce the expression of these cytokines and the activation of these pathways in response to HMGB1, we treated HUVECs with HMGB1 (1 μg/mL, 16 h) followed by CGK012 for 6 h. Notably, our results showed that CGK012 administration suppressed the expression of proinflammatory cytokines and the activation of the NF-κB and ERK signal transduction increased by HMGB1 in endothelial cells (Figure 6A–F). Additionally, CGK012 downregulated the enhanced subnuclear localization of p65 NF-κB induced by HMGB1 in HUVECs (Figure 6G).

### 2.6. Overexpression of HMGB1 Prevents the Anti-Inflammatory Functions of CGK012

**Figure 7:**
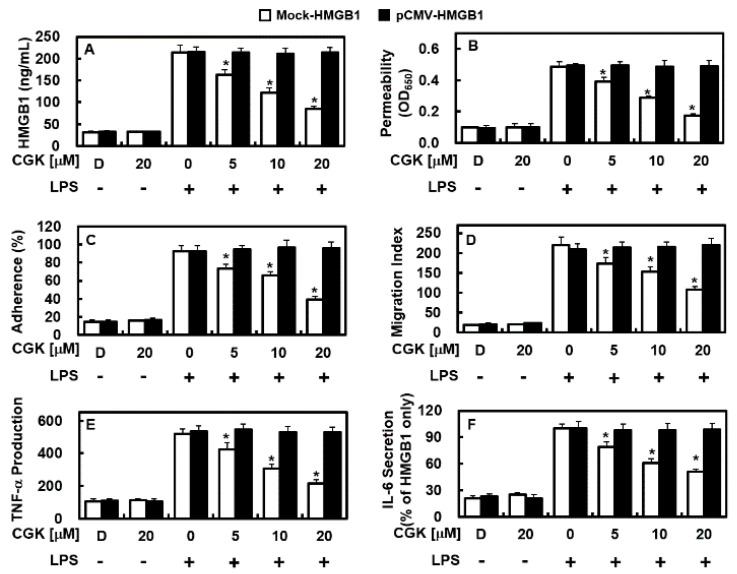
Effects of HMGB1 overexpression on the anti-inflammatory functions of CGK012. Overexpression of human HMGB1 in HUVECs was achieved using the pCMV6-Ac-GFP vector (pCMV-HMGB1). (**A**–**F**) Similar to Figure 2A (**A**), Figure 4A (**B**), Figure 5B (**C**), Figure 5C (**D**), Figure 6A (**E**), and Figure 6B (**F**), except that HUVECs were transfected with mock-vector (Mock-HMGB1) or pCMV6-Ac-GFP vector (pCMV-HMGB1). Data are expressed as the mean ± SD values of three independent experiments on different days. * *p* < 0.05 versus treatment with LPS only.

Next, we conducted an experiment to determine whether HMGB1 overexpression could prevent the anti-inflammatory effects of CGK012. Human HMGB1 was inserted into a vector and introduced into endothelial cells, causing them to overexpress HMGB1. Notably, we found that CGK012 did not suppress HMGB1 expression in cells overexpressing HMGB1 and treated with LPS but was able to suppress HMGB1 expression in cells without HMGB1 overexpression (Figure 7A). Furthermore, the ability of CGK012 to reduce inflammation-related factors, such as TNF-α and IL-6 expression, and the permeability (Figure 7B), adhesiveness (Figure 7C), and translocation of inflammatory immune cells (Figure 7D) were inhibited in cells overexpressing HMGB1. These results suggest that CGK012 was not effective in managing inflammation when HMGB1 was overexpressed, even at higher doses. In addition, HMGB1 overexpression inhibited the ability of CGK012 to suppress TNF-α and IL-6 expression in cells (Figure 7E,F). This indicates that CGK012 was unable to prevent the release of LPS-induced HMGB1 in cells carrying the pCMV6-Ac-GFP vector, which also resulted in the failure of CGK012 to manage lethal inflammation activity induced by LPS, such as the excessive permeability, adherence, and translocation of inflammatory immune cells (Figure 7). These aspects remained difficult for CGK012 even at higher doses. The knockdown or knockout of HMGB1 prevented or protected against HMGB1-induced inflammatory activity in endothelial cells, but other inflammation-related factors could still promote similar inflammatory activity.

### 2.7. Effects of CGK012 Administration on Survival Rate and Tissue Injury Reduction in Mice with CLP-Induced Sepsis

**Figure 8:**
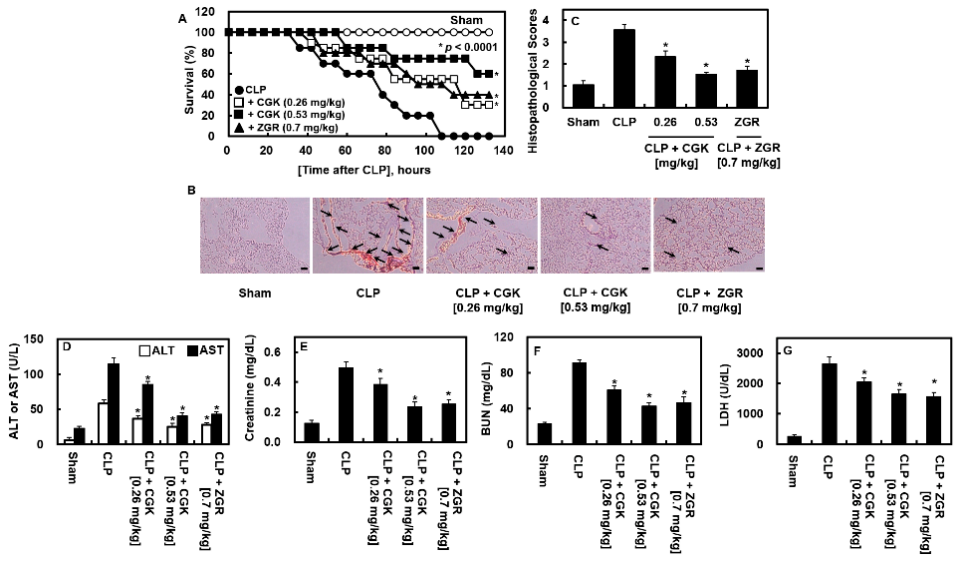
Effects of CGK012 on lethality and tissue injury after the CLP procedure. (**A**) The suppressive effects of CGK012 on mice lethality induced by CLP were monitored every 12 h for 132 h after CLP. Sterile saline was administered to sham-operated mice (○) and control CLP mice (●) (*n* = 20). (**B**) Representative photographs of lung tissues in panel C (H&E staining, 200×); representative images from three independent experiments performed on different days with similar results are shown. Arrows indicate leukocyte infiltration. (**C**) Histological analysis of the effects of CGK012 on CLP-induced lung injury (*n* = 5); histopathological scores of the injured lung tissues were counted and evaluated as described. (**D**–**G**) Suppressive effects of CGK012 on the plasma levels of various organ injury markers: (**D**) aspartate transaminase (AST) and alanine transaminase (ALT), (**E**) creatinine, (**F**) blood urea nitrogen (BUN), and (**G**) lactate dehydrogenase (LDH) (*n* = 20). Data are expressed as the mean ± SD of three independent experiments performed on different days with similar results. * *p* < 0.05 versus CLP.

We administered CGK012 to mice that underwent CLP surgery to investigate its preventive role in the survival of mice with sepsis. Initially, we administered CGK012 or ZGR alone, which did not promote any significant increase in survival rates. Therefore, we administered CGK012 (0.26 or 0.53 mg/kg) or ZGR (0.7 mg/kg) at 12 h and 50 h (for CGK012) or 132 h (for ZGR) after surgery and monitored the mice every 12 h. Mice that received two injections of CGK012 showed a considerable improvement in survival rates after surgery (*p* < 0.00001; Figure 8A). Although CGK012 administration markedly decreased HMGB1 expression once at 12 h after CLP (Figure 2B), our results suggest that two injections were necessary to suppress HMGB1-induced inflammation activity and improve animal survival. Overall, our findings indicate that CGK012 may be a promising candidate for the treatment of sepsis-related pathology. During septic deterioration, acute inflammation can cause multiple organ failure, including critical organs in the pulmonary and renal systems [18]. To investigate the therapeutic effects of CGK012 on CLP-induced pulmonary damage, we examined its activity in lung tissue. We found that CGK012 mitigated the pathological abnormalities caused by CLP and reduced tissue injury (Figure 8B,C). Following CLP surgery, we observed a significant increase in the concentrations of alanine aminotransferase (ALT) and aspartate aminotransferase (AST) in the sera, which indicates liver damage, as well as blood urea nitrogen (BUN) and creatinine, which indicates kidney damage (Figure 8D–F). CGK012 not only decreased the levels of these markers but also reduced the elevation in lactate dehydrogenase (LDH), which is a marker of histological damage (Figure 8G). These results suggest that CGK012 may have potential as a treatment for sepsis-related pathology.

## 3. Discussion

The current study aimed to investigate the therapeutic potential of CGK012 in managing the serious reduction in vascular structure integrity in response to sepsis. The vascular endothelial structure is responsible for maintaining the integrity of the protective vascular layer in healthy cells, with its dysfunction in sepsis causing an acute increase in permeability, resulting in often fatal edema in the body cavity [19]. Hence, managing vascular structure integrity among patients with sepsis is crucial given the critical and influential function of the vascular structure. Pharmacologic approaches that can improve the recovery of the vascular structure and manage vascular consistency hold promise for the treatment of sepsis.

Sepsis remains a critical challenge in healthcare due to its complex pathophysiology and high mortality rates. Existing treatments for sepsis have several limitations that hinder their effectiveness in providing optimal care to patients [2,3,9]. One major limitation is the multifactorial nature of sepsis, which encompasses diverse etiologies, clinical presentations, and underlying mechanisms. This complexity hampers the development of standardized treatment protocols that can effectively address the specific needs of individual patients. Consequently, existing treatments, such as antibiotics and supportive care measures, may not consistently improve outcomes across all septic patients. In addition, delayed diagnosis is another significant limitation in sepsis management. Sepsis can be challenging to diagnose, particularly in its early stages, owing to its nonspecific symptoms and lack of reliable biomarkers [2,3,9]. Delayed diagnosis can lead to deferral of treatment initiation, resulting in poorer patient outcomes. Furthermore, the emergence of antibiotic-resistant pathogens poses a significant challenge in sepsis treatment. Antibiotic resistance compromises the efficacy of standard antibiotic therapies, necessitating alternative treatment strategies. Immune dysregulation, characterized by both hyperinflammation and immunosuppression, is a hallmark of sepsis. Existing treatments may not adequately modulate immune response dysregulation, leading to persistent inflammation, tissue damage, and organ dysfunction. Hence, immunomodulating therapies are needed to restore immune homeostasis and improve outcomes in sepsis. Organ dysfunction is another major challenge in sepsis management. Sepsis can cause multi-organ dysfunction syndrome, in which multiple organ systems fail simultaneously. While supportive care measures such as fluid resuscitation and vasopressor therapy can mitigate organ dysfunction, they do not address the underlying pathophysiology driving organ failure. Targeted therapies that directly address the mechanisms of organ dysfunction are needed to improve outcomes in sepsis [2,3,9].

Targeting HMGB1 represents a promising treatment approach for sepsis given its central role in inflammation and immune dysregulation [20,21]. HMGB1 is a nuclear protein that can be released into the extracellular environment during cellular stress or injury where it functions as a proinflammatory cytokine. HMGB1 plays a critical role in driving the inflammatory response associated with sepsis by activating various receptors and inflammatory pathways [20,21]. By targeting HMGB1, it may be possible to attenuate inflammation, reduce tissue damage, and improve outcomes in sepsis. Studies have explored several strategies for modulating HMGB1 release or activity, including antibodies, small molecule inhibitors, and natural compounds. These approaches have shown promise in preclinical studies by mitigating inflammation, improving organ function, and enhancing survival in experimental models of sepsis [20,21]. While the clinical translation of HMGB1-targeted therapies is still in its early stages, preliminary evidence from clinical studies suggests that targeting HMGB1 may have therapeutic potential in sepsis. Patients with sepsis have high levels of HMGB1, which have been associated with poor outcomes. Hence, targeting HMGB1 may modulate the inflammatory response and improve outcomes in patients with sepsis [20,21].

There are two ways to decrease the molecular activities responsible for the release of HMGB1, namely passive and active methods [8,20]. Passive secretion is caused by cell death, either programmed or necrotic, whereas active secretion is induced by posttranslational modification of nuclear HMGB1 [8,20]. Previous studies [17,22] indicate that the acetylation of lysine residues within specific intranuclear regions of HMGB1 is important for the posttranslational modification response to pathogenic antigens, like LPS. Our experiments showed that LPS promoted HMGB1 secretion (Figure 2A) and lysine residue acetylation (Figure 3B), indicating that HUVECs release HMGB1 through the active method.

Based on the current finding that CGK012 significantly reduces HMGB1 secretion in LPS-stimulated HUVECs via the SIRT1-related deacetylation of HMGB1 and HMGB1-induced barrier integrity reduction, leakage, and phosphorylated p38 by CGK012, we conclude that the observed modulation of SIRT1/HMGB1 by CGK012 appears to play a crucial role in maintaining vascular barrier integrity by regulating HMGB1 acetylation and secretion. By enhancing the SIRT1-mediated deacetylation of HMGB1, CGK012 may mitigate the damaging effects of HMGB1 on the vascular barrier, highlighting its therapeutic potential in pathological vascular inflammation associated with conditions such as sepsis.

The ability of CGK012 to prevent the progression of HMGB1-induced sepsis was examined by investigating its effects on HMGB1 secretion (Figure 2A,B); the expression of HMGB1 signal receptors, such as TLR2, TLR4, and RAGE (Figure 2C); and the HMGB1-induced increase in permeability (Figure 4A–C) by blocking the p38 biochemical modification (Figure 4D) and its pathway. Additionally, CGK012 was observed to downregulate the adhesion molecules (E-selectin, VCAM, and ICAM) that participate in the reaction between inflammatory immune cells and endothelial cells (Figure 5). Furthermore, the administration of BIMP, which inhibits HMGB1 release, decreased the levels of proinflammatory cytokines such as TNF-α, β, IL-6, and IL-1β (Figure 6A–C), reduced the response of transcription factors NF-κB and ERK1/2 (Figure 6E,F) in inflammation, and decreased the transfer of NF-κB to the extranuclear matrix (Figure 6G). The protective action of CGK012 inhibited these HMGB1-mediated modifications and HMGB1 itself, as well as other mediators involved in the inflammatory signaling pathway.

During sepsis, the NF-κB response of the endothelial structure is responsible for the activation of several pathways leading to hyperpermeability, such as cytokine, chemokine, and enzyme production and CAM elevation regulated by NF-κB [23,24]. Suppressing the NF-κB pathway, particularly in the endothelium, may protect against inflammation [25]. The reduction of CAMs and other inflammation-related factors in the vascular barrier by inhibiting NF-κB can prevent the induction of inflammatory immune cells and alleviate the symptoms of sepsis [26,27].

Developing organic synthetic compounds into drugs involves several challenges and limitations that researchers must address [28]. One limitation is the complexity of the drug development process itself, which typically involves multiple stages, including compound identification, preclinical testing, clinical trials, regulatory approval, and post-marketing surveillance. Each stage requires a significant amount of time, resources, and expertise, making the overall process lengthy and expensive. Another limitation is the unpredictability of drug efficacy and safety. Even promising compounds identified through preclinical studies may fail to demonstrate efficacy or safety in clinical trials due to various factors, such as pharmacokinetic variability, off-target effects, and patient heterogeneity. Additionally, the development of drug resistance, especially in the case of antimicrobial agents, poses a significant challenge [28]. To address these limitations and improve the process of developing organic synthetic compounds into drugs, future plans may integrate innovative technologies and methodologies. This could include the use of computational modeling and artificial intelligence to streamline the drug discovery process, identify promising drug candidates, and predict their efficacy and safety profiles more accurately. Moreover, advancements in personalized medicine and precision therapeutics may enable the development of targeted treatments tailored to individual patient characteristics, thereby improving treatment outcomes and reducing adverse effects. Collaboration between academia, industry, and regulatory agencies will also be essential in accelerating drug development timelines, enhancing research efficiency, and facilitating regulatory approval processes [28].

CGK012 can effectively counter the malignancy induced by HMGB1 in the vascular structure by reducing the secretion of HMGB1 in LPS-stimulated endothelial cells, inhibiting the CLP-induced secretion of HMGB1, and improving vascular wall stability. The restorative effects of CGK012 on the vascular wall structure were observed in mice that underwent CLP, with CGK012 treatment improving their survival rate and reducing tissue/organ injuries. The current study has been the first to show that CGK012 potently inhibits sepsis through multiple effects on inflammation-related factors in vitro and in vivo. These results indicate that a pyranocoumarin and cyclopentyl carbamate, the chemical composition of CGK012, is required to inhibit sepsis. We believe that our findings will provide important information for the development of drug candidates for sepsis, including structure–activity relationship (SAR) studies, and facilitate research on the relationship between Wnt/β-catenin signaling and sepsis.

## 4. Materials and Methods

### 4.1. Cell Culture and Reagents

Cambrex Bio Science (Charles City, IA, USA) produced primary HUVECs using established methods [29,30]. For all experiments, cells at passage 3–5 were used. Human neutrophils were freshly isolated from whole blood (15 mL) obtained from five healthy volunteers via venipuncture and maintained as previously described [31,32]. Positive control for anti-inflammatory effects was achieved using ZGR (purity > 96%) at 0.7 mg/kg for in vivo assays and 20 μM for in vitro assays [13,14]. LPS, crystal violet, Evans blue, MTT, 2-penicillin G and streptomycin, mercaptoethanol, and dimethyl sulfoxide (DMSO) were obtained from Sigma Chemical Co. (St. Louis, MO, USA). Genetically processed human HMGB1 was obtained from Abnova (Taipei City, Taiwan). A vehicle control and 0.5% DMSO were used for dissolving CGK012 (Figure 1) or ZGR for use. For the in vitro and in vivo assays, CGK012 was used at different doses (1, 2, 5, 10, or 20 μM and 0.03, 0.05, 0.13, 0.26, or 0.53 mg/kg, respectively). Based on a total blood volume of 72 mL/kg [33,34,35] and an average individual mass of 27 g per mouse in the experiment, the total average blood volume was determined to be 2 mL. After calculations for dilution in peripheral blood, CGK012 (0.05, 0.13, 0.26, or 0.53 mg/kg) was administered at concentrations of 2, 5, 10, or 20 µM.

### 4.2. Method for the Synthesis of (7S)-(+)-Cyclopentyl Carbamic Acid 8,8-Dimethyl-2-oxo-6,7-dihydro-2H,8H-pyrano[3,2-g]chromen-7-yl-ester (CGK012)

**Scheme 1:**
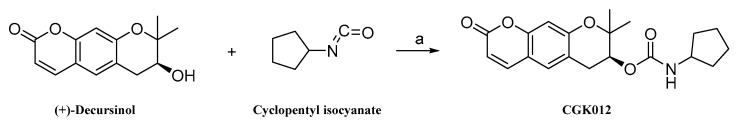
Synthesis of CGK012. Reagents and conditions; (a) triethylamine (1.5 eq), 4-dimethylaminopyridine (0.6 eq), toluene, 110 °C, 24 h.

The method for synthesizing CGK012 is depicted in Scheme 1. Briefly, (+)-decursinol (1 g, 4.06 mmol, 1 eq) was added to a solution of cyclopentyl isocyanate (0.67 g, 6.09 mmol, 1.5 eq), triethylamine (0.74 g, 7.31 mmol, 1.8 eq), and 4-dimethylaminopyridine (0.29 g, 2.44 mmol, 0.6 eq) in anhydrous toluene (300 mL). The reaction mixture was stirred for 24 h at 110 °C. After detecting a complete reaction by TLC, the mixture was cooled at room temperature, filtered on celite, and washed with toluene. The combined organic layers were washed three times with 300 mL of 3N HCl aqueous solution. The acid-treated organic layers were washed with 300 mL of saturated K_2_CO_3_ aqueous solution and 300 mL of water. The combined organic layers were dried over anhydrous MgSO4, filtered, and evaporated under vacuum conditions. The resulting crystalline solid was washed with n-pentane and filtered to obtain the CGK012 with the following characteristics: yield 98.4%; ivory-white solid; mp 162 °C; [α]$\frac{25}{D}$ + 78.08 (c = 0.1. MeOH); 1H NMR (600 MHz, DMSO): δH 7.92 (1H, d, *J* = 9.5 Hz, 4-H), 7.48 (1H, s, 5-H), 7.24 (1H, d, *J* = 7.1 Hz, 7-H), 6.76 (1H, s, 10-H), 6.26 (1H, d, *J* = 9.5 Hz, 3-H), 4.91 (1H, t, *J* = 3.9 Hz, 2′-NH), 3.77 (1H, dd, *J* = 13.7, 6.9 Hz, 3′-H), 3.19 (1H, dd, *J* = 17.6, 4.1 Hz, 6-H), 2.82 (1H, dd, *J* = 17.5, 3.3 Hz, 6-H), 1.74 (2H, dt, *J* = 12.4, 5.3 Hz, 7′-H), 1.57 (2H, s, 4′-H), 1.44 (2H, d, *J* = 6.8 Hz, 6′-H), 1.34 (5H, s, 5′-H, 8CH3-H), 1.29 (3H, s, 8CH3-H); 13C NMR (151 MHz, DMSO): δC 160.23 (C-2), 156.03 (C-9a), 155.10 (C-1′), 153.53 (C-10a), 144.07 (C-4), 129.56 (C-5), 116.17 (C-5a), 112.62 (C-4a), 112.48 (C-3), 103.41 (C-10), 77.12 (C-8), 68.85 (C-7), 52.11 (C-3′), 32.20 (C-7′), 32.04 (C-4′), 27.51 (C-6), 24.49 (C-8CH3), 23.49(C-8CH3), 23.15 (C-5′, C-6′); C20H23NO5 [M+H]^+^ at *m*/*z*; 358.2. The purity (99.731%) of CGK012 was confirmed using HPLC analysis (Shimadzu, Kyoto, Japan, detector 254 nm, column; shim-pack GIS-ODA [150 × 4.6 mm, 5 μm]), column oven 40 °C, flow rate 1.0 mL/min, mobile phase (H_2_O:ACN = 6:4)].

### 4.3. Animals and CLP

Male C57BL/6 mice (6–7 weeks old; weight, 27 g) were obtained from Orient Bio Co. (Sungnam, Republic of Korea). The CLP mouse model was created following a previously published protocol [35,36]. The sham group underwent cecal exposure without ligation or puncture. The samples were intravenously injected with CGK012 at doses of 0.05, 0.13, 0.26, or 0.53 mg/kg or 0.7 mg/kg ZGR 24 h after CLP surgery. Thereafter, we analyzed HMGB1 release, cell integrity, and immune cell migration. To assess survival rate, samples were intravenously injected with 0.26 or 0.53 mg/kg of CGK012 or 0.7 mg/kg of ZGR 12 and 50 h after CLP (Figure 8). The Animal Care Committee at Kyungpook National University approved these animal experiments before experimentation (IRB No. KNU 2021-104).

### 4.4. Competitive ELISA for HMGB1

To determine the release of HMGB1 in mouse sera or cell culture fluid, a competitive ELISA was conducted following previously described methods [37,38].

### 4.5. Expression Levels of CAMs and HMGB1 Receptors

Levels of VCAM-1, ICAM-1, and E-selectin in HUVECs were analyzed using whole-cell ELISAs conducted according to previously described methods [13]. Briefly, HUVECs were treated with HMGB1 (1 μg/mL) for either 16 h (to assess VCAM-1 and ICAM-1 levels) or 22 h (to assess E-selectin levels) after they had formed a single layer at 80–90% confluence. Following HMGB1 stimulation, CGK012 treatment or ZGR administration was performed. Antibodies A-9, H-80, and A-9 (obtained from Santa Cruz Biotechnology Inc., Dallas, TX, USA) were used to measure the expression levels of TLR2, TLR4, and RAGE.

### 4.6. Cell Viability Assay

The viability of cultured HUVECs was estimated by performing the MTT assay after incubation with CGK012 for 48 h, as previously described [37,38].

### 4.7. Preparation of Cytoplasmic and Nuclear Extracts and Western Blotting Analyses

To obtain the cytoplasmic and nuclear fractions from cell pellets stored on ice, centrifugation, immunoprecipitation, and Western blotting were conducted as previously described [39]. The loading controls for Western blotting analysis of the cytoplasmic and nuclear fractions were actin and lamin B antibodies. Concentration analysis of the acquired data was conducted using the ImageJ (ver 1.53e) Gel Analysis tool provided by the National Institutes of Health (Bethesda, MD, USA).

### 4.8. Cell–Cell Adhesion Assay

To assess the adhesion between human neutrophilic leukocytes and HUVECs [13], each HUVEC layer was stimulated with HMGB1 (1 μg/mL) for 16 h and then incubated with either 20 µM ZGR or increasing concentrations of CGK012 for 6 h. Neutrophilic leukocytes (3 × 10^5^ cells/well) were stained with Vybrant DiD dye and then coated with a layer of activated HUVECs. The amount of stained coated cells was measured spectrophotometrically for fluorescence emission (total signal) of Vybrant DiD dye using a microplate reader (Tecan, GmbH, Grödig, Austria). After incubating neutrophilic leukocytes and HUVECs for 1 h, non-adherent cells were removed through gentle rinsing with PBS four times. Thereafter, the fluorescence emission from the remaining cells was measured again (i.e., the adherent signal). The proportion of neutrophilic leukocyte adhesion to HUVECs was determined using the following formula:

% adherence = (adherent signal/total signal) × 100.

### 4.9. Permeability Assay

To assess the effects of CGK012 and ZGR on cell permeability in vitro, a bi-compartmental chamber was used as previously described [13]. In vivo, male mice were given HMGB1 (2 μg/mouse) via intravenous injection for 16 h, followed by an injection of either CGK012 compound (0.05, 0.13, 0.26, or 0.53 mg/kg) or ZGR (0.7 mg/kg). The amount of Evans blue dye injected intravenously and leaked into the abdominal cavity per mouse was measured and a standard curve was used to estimate the precise flux through the vascular structure, as detailed in a previous study [13].

### 4.10. In Vitro Migration Assay

The level of migration of human neutrophilic leukocytes through HUVECs was determined following a previously described protocol [13]. The experiment was conducted using 6.5 mm Transwell plates with an 8 µm pore size filter. HUVECs were incubated for 72 h to obtain a fully confluent monolayer of endothelial cells. Following stimulation with HMGB1 (1 μg/mL) for 16 h and treatment with ZGR or increasing concentrations of CGK012 for 6 h, neutrophilic leukocytes were added to the upper chamber of the Transwell plate. After 2 h of incubation at 37 °C, non-adherent and non-migrating cells were removed, and the translocated neutrophilic leukocytes were fixed with 8% glutaraldehyde and stained with 0.25% crystal violet in 20% methanol (*w*/*v*). The number of cells in nine randomly selected microscopic fields (200×) was counted, and the assay was repeated twice per well on duplicate wells under the same conditions. The results were reported as the extent of migration.

### 4.11. In Vivo Leukocyte Migration Assay

Mice used in the in vivo experiment were anesthetized using a combination of 2% isoflurane in oxygen delivered through an inhaling chamber and a facemask attached to veterinary anesthesia equipment for small rodents. Leukocyte translocation was then assessed in vivo, following a previously described protocol [12,40]. The mice received an intravenous injection of HMGB1 (2 μg/mouse), followed by an infusion of CGK012 (0.05, 0.13, 0.26, or 0.53 mg/kg) or ZGR (0.7 mg/kg) after 16 h. Thereafter, the mice were euthanized through cervical dislocation, and their abdominal cavity was rinsed with PBS (5 mL). The collected liquid samples (20 μL) were stained with Turk’s solution (0.01% crystal violet in 3% acetic acid), and the cell count was determined using an optical microscope.

### 4.12. ELISAs for Phosphorylation of p38 MAPK/SAPK, NF-κB, TNF-α, ERK 1/2, IL-1β, and IL-6

The level of activation of p38 mitogen-activated protein kinase/stress-activated protein kinase (MAPK/SAPK) was determined by measuring the amount of phosphorylated p38 MAPK/SAPK using an ELISA kit (Cell Signaling Technology, Danvers, MA, USA). The expression levels of IL-1β, IL-6, and TNF-α in the cell culture supernatants, as well as the total and phosphorylated levels of extracellular signal-regulated kinase (ERK) 1/2 (R&D Systems, Minneapolis, MN, USA) and the p65 subunit of nuclear factor kappa B (NF-κB) in the nuclear lysates, were measured using ELISA kits.

### 4.13. Transfection for Stable Human HMGB1-Overexpressed HUVECs

To increase the amount of HMGB1 in HUVECs, a human HMGB1 gene was inserted into a pCMV6-Ac-GFP vector (RG205918, OriGene Technologies, Inc., Rockville, MD, USA). HUVECs were then plated into a 12-well plate at 50%–60% confluency and incubated with 5 µg plasmid DNA using Lipofectamine 3000 (Invitrogen, Carlsbad, CA, USA) in accordance with the manufacturer’s protocols. The cells were then cultured in a medium containing neomycin (4 mg/mL) to ensure stable and safe DNA insertion. After 3 weeks, cells that demonstrated neomycin resistance were observed.

### 4.14. H&E Staining and Histopathological Examinations

For the animal model of sepsis, male mice who underwent CLP were intravenously injected with CGK012 (at a dose of 0.26 or 0.53 mg/kg) or ZGR (at a dose of 0.7 mg/kg) 12 and 50 h after surgery. The mice were euthanized after 4 days, and a blinded analysis of lung tissue histology was performed using an optical microscope. The analysis included examination of the vascular structure, immune cell migration, and tissue edema, which were previously described by Lee et al. [12].

### 4.15. Measurement of Tissue Injury Markers

Commercially available products were used to detect the plasma concentrations of BUN, creatinine, ALT, AST, and LDH.

### 4.16. Statistical Analysis

The results were reported as mean values with standard deviations (SD) based on three independent experiments, unless otherwise stated. To compare the data among different groups, ANOVA and Tukey’s post-hoc analysis were employed. *p* values < 0.05 indicated statistical significance. The Kaplan–Meier curve was utilized to determine the disparity in the survival rates between mice in the CLP model and the control group.

## Data Availability

The data presented in this study are available upon reasonable request from the corresponding author.
